# The Flavoprotein Dodecin as a Redox Probe for Electron Transfer through DNA**

**DOI:** 10.1002/anie.201208987

**Published:** 2013-03-26

**Authors:** Yaming Yu, Björn Heidel, Tamara Lourdes Parapugna, Sabine Wenderhold-Reeb, Bo Song, Holger Schönherr, Martin Grininger, Gilbert Nöll

**Affiliations:** NRW Nachwuchsforschergruppe für Nanotechnologie, Organische Chemie, Universität SiegenFakultät IV, Department für Chemie und Biologie, Adolf-Reichwein-Strasse 2, 57076 Siegen (Germany) E-mail: noell@chemie.uni-siegen.de Homepage: http://www.chemie-biologie.uni-siegen.de/oc/oc1/gruppe_noell/index.html; Physikalische Chemie I, Universität Siegen, Fakultät IV, Department für Chemie und BiologieAdolf-Reichwein-Strasse 2, 57076 Siegen (Germany); Institut für Organische Chemie und Chemische Biologie, Buchmann Institut für Molekulare Lebenswissenschaften, Cluster of Excellence “Macromolecular Complexes”, Goethe Universität FrankfurtMax-von-Laue-Strasse 15, 60438 Frankfurt am Main (Germany)

**Keywords:** DNA, dodecin, electron transfer, flavin, surface plasmon resonance

Dedicated to Prof. Bernhard Dick on the occasion of his 60th birthday

Nowadays it is well established that photochemically induced electron transfer (ET) through DNA in solution is limited to relatively short distances. In most experimental studies, photochemically induced ET was observed over less than 10 base pairs (bp; 3.4 nm),^1^ and, because ET is dependent on the base sequence,1h,i,m at shorter distances (under 5 base pairs) ET is not always efficient. The situation is different for electrochemically induced ET, that is, when ET through redox-labeled DNA monolayers adsorbed on gold electrodes by thiol linkers is investigated. ET over distances of up to 100 base pairs (34 nm) has been reported recently, implying that DNA behaves as a molecular wire.^2^ Since in these studies ET is believed to occur through the π-stack of fully base-paired double-stranded DNA (ds-DNA), applications in biosensing to trace single base mismatches are anticipated.^2^, ^3^ However, the corresponding experimental studies are based solely on cyclic voltammetry, and even the ET rates were determined from the shape of the cyclic voltammograms (CVs).^2^–^4^ Therefore it is of interest to develop alternative experimental approaches to study electrochemically induced ET through DNA monolayers.

Herein we use the riboflavin binding protein dodecin as a redox-sensitive probe to study ET processes through DNA monolayers. The ET signal is translated into a change of surface-bound mass, thus allowing further analytical techniques, such as surface plasmon resonance (SPR) or quartz crystal microbalance (QCM) measurements, to be used to trace ET. Dodecin from *Halobacterium salinarum* is a hollow spherical dodecameric protein, which binds oxidized flavins with high affinity, whereas flavin reduction induces the dissociation of the holoprotein.^5^ To study ET, dodecin can be reconstituted on flavin-terminated ds-DNA adsorbed on gold electrodes. If ET through DNA is possible, applying a negative potential will result in flavin reduction and subsequent release of apododecin. In a first study, ET through DNA 20 base pairs long was not observed.5b,f Since rather long saturated tethers between gold electrode and DNA were used, which could have been a bottleneck for ET, we have now minimized the ET distance between electrode and DNA. The experimental approach used to show whether ds-DNA of 20 base pairs is suitable for application as a molecular wire is depicted in Scheme 1 (for experimental details see Supporting Information).

**Scheme 1:**
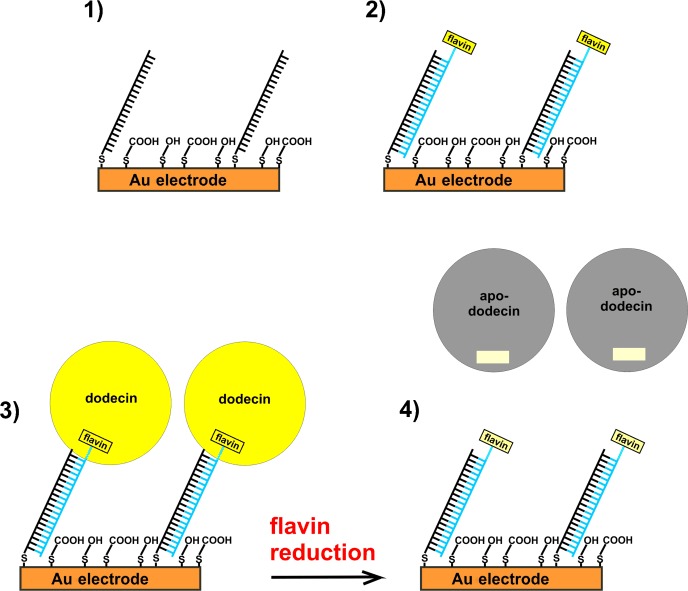
Experimental approach using dodecin to probe electron-transfer through DNA. 1) A monolayer of disulfide modified ss-DNA is adsorbed on gold whereupon a mixture of mercaptobutanol (MCB) and mercaptopropionic acid (MPA) in a ratio of 1:1 is added to release non-specifically adsorbed DNA and to increase the hybridization efficiency. 2) Complementary flavin-modified ss-DNA is hybridized. 3) Dodecin is reconstituted at the surface. 4) A negative potential is applied. If electron transfer through ds-DNA is possible, this results in flavin reduction followed by the release of apododecin (only one of the six flavin binding pockets of dodecin is shown).

For the adsorption of disulfide-modified ss-DNA onto gold, three 1,2-dithiane rings forming six gold–sulfur bonds are used (see Scheme 2). With this linkage the DNA is bound stronger,^6^ and the ET distance between the gold surface and the first DNA base is shorter than with the commonly used hexylthiol linker.^2^, ^3^, 5b The DNA sequence is chosen in a way that an unambiguous hybridization product is formed. Prior to adsorption of apododecin tE, a non-binding dodecin variant dA was added as a negative control to show that apododecin tE is bound specifically to the flavins.5b To minimize the ET distance between the redox-active isoalloxazine subunit of flavin and DNA, different flavin–DNA ligands comprising only the first five bases at the 5′ end (oligo 5, O5) and flavins of different lengths (CofC*n*) were synthesized. Dodecin was reconstituted with these ligands and X-ray structures were measured. In Figure 1 A the structures of the flavin–DNA ligands CofC*n* O5 with a hexyl, butyl, propyl, and ethyl group (*n*=6, 4, 3, 2) at the N(10) of the isoalloxazine moiety are shown. For all the flavin–DNA ligands, electron density can be traced for the isoalloxazine moiety (and the first two CH_2_ groups). For the CofC4_O5 ligand, additional electron density is present, probably caused by weak binding of the linker and the first DNA base. For the CofC2_O5 ligand further electron density indicates binding between apoprotein and linker plus the first two DNA bases. As shown by superposition of the ligands in Figure 1 B, the (rather deep) position of the isoalloxazine ring of CofC2_O5 in the binding pocket is similar to that of lumiflavin, while all the other flavin–DNA hybrid ligands superimpose with riboflavin. This result is surprising, as CofC2_O5 was found to bind 25-times weaker than CofC4_O5 (*K*_d_ of 10 μm vs. 400 nm), but finds a deeper position, and previously a deeper position has been correlated with a higher binding affinity.5e However, as depicted in the side view of Figure 1 B, the isoalloxazine of CofC2_O5 is tilted, and it is tempting to speculate that the tilted incorporation of the isoalloxazine causes lower affinity.

**Figure 1:**
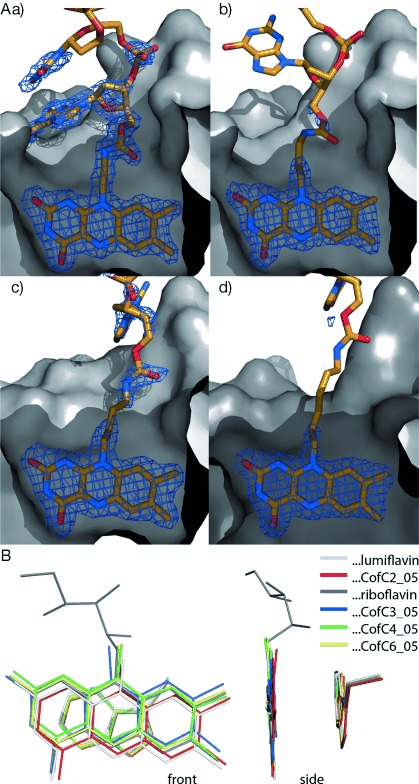
X-ray structures of CofC*n*_O5 flavin–DNA ligands in the tE dodecin binding pocket. Dodecin is depicted as apoprotein in a surface representation (gray) with the incorporated ligands shown in stick representation (C orange, N blue, O red). Dodecin has six binding pockets for the incorporation of dimers of flavins in a *C*_2_-symmetric manner. For clarity the *C*_2_-symmetric part of one binding pocket is shown. Structural data used for figures are deposited with the protein data bank (pdb) codes 2vkg, 2vkf, and 4b2h (http://www.rcsb.org). A) tE dodecin with bound CofC*n*_O5 ligands with varying lengths of alkyl chain connecting isoalloxazine to DNA. CofC2_O5 (a) has an ethyl chain, CofC3_O5 (b) propyl, CofC4_O5 (c) butyl, and CofC6_O5 (d) hexyl. Electron omit density is shown at *σ*=1.5 as a blue mesh, highlighting protein-bound substructures of ligands. The unbound parts of the ligands, which could not be identified by electron density, are shown for clarity in one possible conformation. (B) Superposition of Cα-backbone-aligned tE dodecins with CofC*n*_O5 ligands (and tryptophan, which is part of the binding site) in front and side view. Ligands are depicted by their isoalloxazine anchor. Wildtype dodecin with riboflavin and lumiflavin ligands are shown for comparison.

**Scheme 2:**
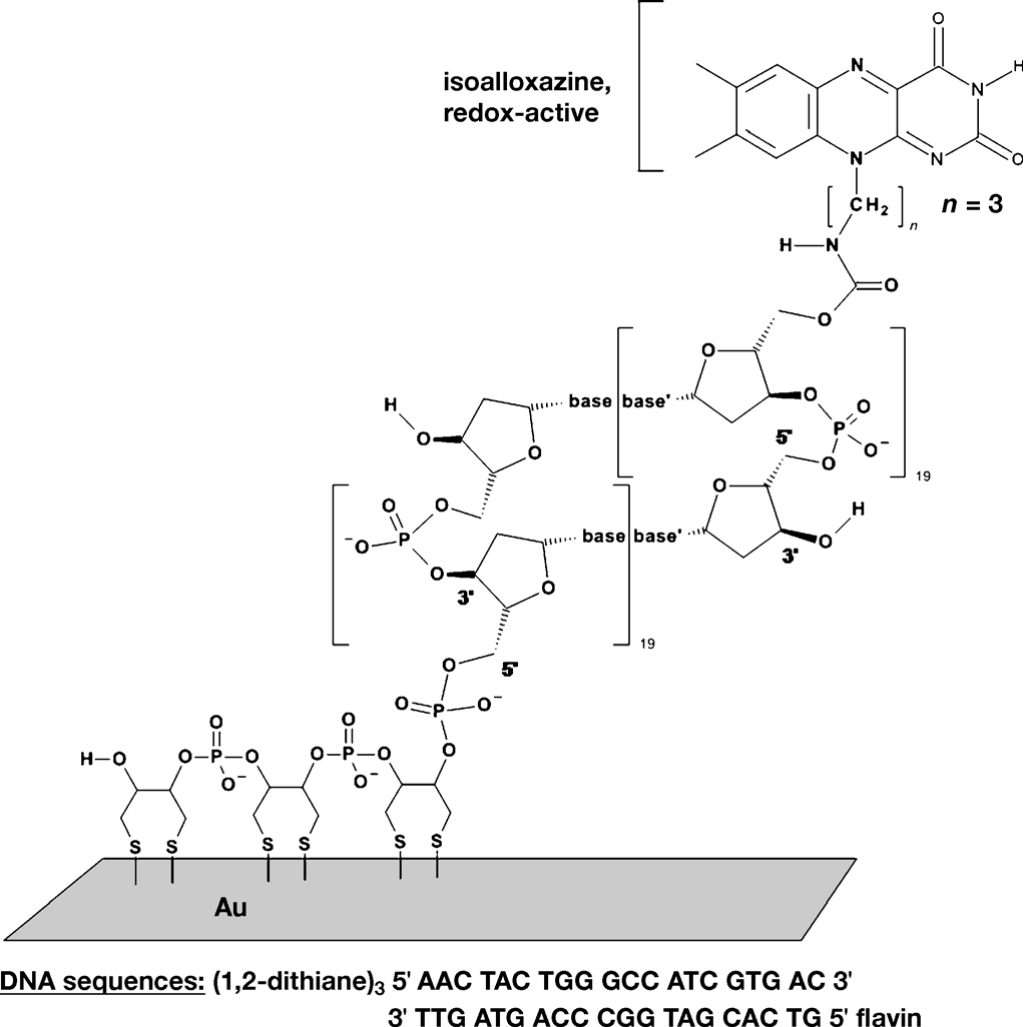
Structure and sequence of the flavin- and disulfide-modified DNA.

For electrochemical measurements a ligand with *n*=3, bound only by its (oxidized) isoalloxazine moiety, appeared to be the best compromise between high affinity binding and short ET distance. Dodecin was reconstituted in a flow cell for combined electrochemistry and SPR measurements. The SPR kinetic scans collected during the stepwise reconstitution of dodecin on the flavin terminated ds-DNA monolayer and subsequent electrochemical experiments are shown in Figure 2. After formation of a dodecin monolayer as outlined in Scheme 1 (1–3) we changed to an argon-saturated buffer and applied a negative potential (up to −650 mV vs. Ag/AgCl) but no release of tE was observed. In addition cyclic voltammograms (CVs) were collected at different scan rates before and after dodecin reconstitution, but in none of the CVs could the flavin reduction be detected.

**Figure 2:**
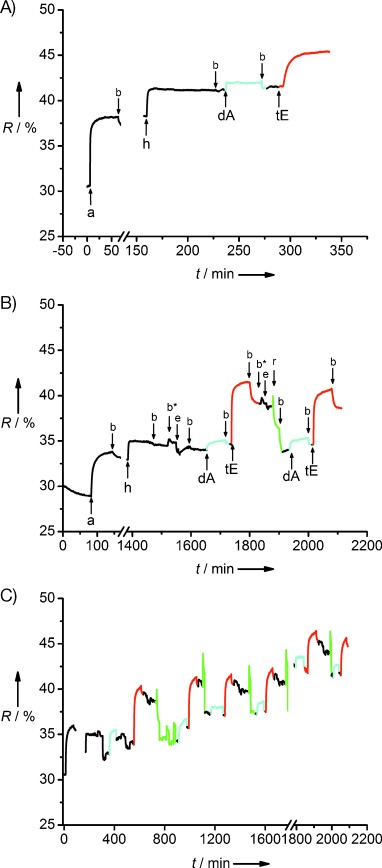
Kinetic SPR scan curves A) adsorption of disulfide modified ss-DNA (a), rinsing with buffer (b), hybridization with flavin-modified ss-DNA (h), incubation of non-binding apododecin as negative control (dA), reconstitution of dodecin (tE). During addition of MCB/MPA in water the reflectivity *R* changed to values below 10 %, because of the difference in reflective index between water and buffer (not shown). B) The experimental procedure was extended by the following steps: rinsing with argon saturated buffer (b*), electrochemistry, that is, application of −550 mV vs. Ag/AgCl for 5 min then cyclic voltammetry (e), chemical reduction using sodium dithionite in argon-saturated buffer (r). C) Long-term run. Incubation of dA and rinsing with buffer is shown in cyan, incubation of tE and rinsing with buffer is shown red, reduction and rinsing is shown in green.

To demonstrate that the release of tE upon reduction is indeed possible, a chemical reduction step was carried out by addition of sodium dithionite (see Figure 2 B). By chemical reduction and subsequent rinsing with buffer, it was possible to release tE almost quantitatively. Thereafter a negative control was carried out, by adding non-binding dA. On subsequent addition of tE, dodecin was reconstituted again, demonstrating that the flavin-terminated ds-DNA monolayer was still intact. The entire experiment was carried out three times leading to the same results. To examine the long-term stability of the system, an extended experimental run was carried out (see Figure 2 C and Figure S1 in the Supporting Information). After tE was adsorbed and released four times by chemical reduction, the flow cell was stored in the fridge at 8 °C for five days, before the experiment was continued. After this break, it was still possible to bind and release tE as before.

We further characterized a reconstituted dodecin monolayer on gold by atomic force microscopy (AFM). For this purpose dodecin was reconstituted as before on a template stripped gold (TSG) slide with flat surface.^7^ The resulting images are depicted in Figure 3. After adsorption of dodecin, the surface was covered by densely packed round features with an average lateral dimension of about (12.5±4) nm (Figure 3, left). The size of dodecin was measured without correction for AFM tip broadening. The real size after correction is about half of the (12.5±4) nm or even smaller. This corrected data is similar to the actual size of dodecin (6–7 nm) and the round features are attributed to individual dodecin molecules. To show that we can control the density of dodecin at the surface, and the elevated protrusions in Figure 3 are indeed reconstituted dodecin molecules, we reduced the flavin density (by decreasing the concentration of disulfide and flavin modified ss-DNA by a factor of 10). As shown in Figure 3, right, a lower density of the features attributed to reconstituted dodecin was obtained. Owing to the established tip broadening effect, the apparent lateral dimensions of the elevated protrusions appear to be slightly broader than in the left panel, but were in the same range as before.

**Figure 3:**
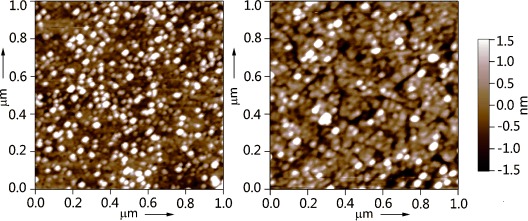
AFM images (acquired in air) of the dodecin reconstituted on the flavin terminated ds-DNA monolayer adsorbed on template-stripped gold prepared using the same concentrations for DNA adsorption and hybridization as for SPR measurements (left) and lower flavin concentration (right).

While DNA-mediated charge transfer between DNA repair proteins has been proposed as a long-range signaling mechanism to trace damages in the genome,^8^ in the current study DNA covalently bound in between a flavin/flavoprotein and a gold electrode did not mediate ET, even though the distances electrode–DNA and DNA–redox-center were minimized. Efficient ET could be observed even for larger distances between isoalloxazine and “molecular wires”, when the flavoenzyme glucose oxidase was reconstituted on gold electrodes, but using molecules other than DNA (e.g. single-walled carbon nanotubes) as the molecular wire.5f, 9 In the X-ray structures there was no electron density for most of the linker between DNA and flavin indicating that (also after protein reconstitution) different orientations of DNA and redox-center relative to each other are possible. Nevertheless, we did not detect any ET through DNA implying that ds-DNA (of 20 base pairs or more) is not suitable for an application as molecular wire. This is in contrast to recent reports based solely on cyclic voltammetry data.^2^, ^3^ However, the fact that a redox system attached to DNA can be reduced by cyclic voltammetry does not necessarily mean that ET occurs along the DNA base pairs.5b, 10 In future studies using dodecin–DNA we will investigate whether ET can be enabled by reducing the number of base pairs and/or varying the sequence.

## Supplementary material

As a service to our authors and readers, this journal provides supporting information supplied by the authors. Such materials are peer reviewed and may be re-organized for online delivery, but are not copy-edited or typeset. Technical support issues arising from supporting information (other than missing files) should be addressed to the authors.
