# Supplementary material for: The Flavoprotein Dodecin as a Redox Probe for Electron Transfer through DNA
Source: Angew Chem Int Ed Engl. 2013 Mar 26;52(18):4950–3. doi: 10.1002/anie.201208987 (PMC3743158; doi:10.1002/anie.201208987)
Supplement: Supplementary file 1 [file anie0052-4950-SD1.pdf]

Angewandte Chemie
Eine Zeitschrift der Gesellschaft Deutscher Chemiker

Supporting Information

© Wiley-VCH 2013

69451 Weinheim, Germany

The Flavoprotein Dodecin as a Redox Probe for Electron Transfer through DNA**

*Yaming Yu, Björn Heidel, Tamara Lourdes Parapugna, Sabine Wenderhold-Reeb, Bo Song, Holger Schönherr, Martin Grininger, and Gilbert Nöll**

anie_201208987_sm_miscellaneous_information.pdf

Supporting information

Table of contents

i) Experimental Section (Detailed information about materials, gold slide preparation, surface modification, X-ray structural analysis, and instrumentation is given.)

ii) Table S1. X-ray Data collection and refinement statistics

iii) SPR long-term run (kinetic scan curve) depicted in Figure S1 including a detailed description of the individual experimental steps (shown previously as Figure 2C in the main manuscript)

iv) Optical thickness of the dodecin monolayers

i) Experimental Section

Materials. 4-mercapto-1-butanol (MCB) and 3-mecaptopropionic acid (MPA) were purchased from Sigma-Aldrich. Tris-base and sodium dithionite were obtained from Alfa Aesar. The nonbinding and binding dodecin variants were synthesized as reported previously.[1] The flavins were synthesized and linked to DNA as published previously.[1] The modified DNA was purchased from *Eurogentec*, Seraing, Belgium.
Solution preparation. All solutions were prepared with Milli-Q water (18 MΩcm, Millipore) unless stated otherwise. As buffer, 20 mM Tris-HCl, 1M NaCl, 5mM $MgCl_2$ (pH = 7.5) was used throughout the experiments.

Gold slide preparation. Gold substrates were prepared by vacuum evaporation onto cleaned glass substrates precoated with titanium to improve adhesion (typically, 50 nm of Au, 1.5 nm of Ti). The gold evaporated substrates were freshly cleaned prior to use by the treatment with a piranha solution (consisting of 70% concentrated sulfuric acid and 30% hydrogen peroxide, CAUTION: piranha solution reacts violently with most organic materials and must be handled with extreme care) for 5 min, and then thoroughly rinsed with pure water. Template-stripped gold (TSG) substrates with smooth surfaces were prepared as described elsewhere.[2] Briefly, gold films with thickness of 50 nm were deposited by vacuum evaporation on silicon wafers. Gold surfaces were then glued with EPO-TEK 377 to glass substrates and cured for 60 min at 150 °C. After cooling, the slides were detached from the silicon wafer sheets to expose the TSG film and modified directly.

Surface modification. The flavin terminated DNA monolayer was formed in a flow cell by treating the gold substrate with a 8 µM solution of $(1,2\text{-dithiane})_3$ 5' ss-DNA (sequence: AAC TAC TGG GCC ATC GTG AC) in Tris-HCl buffer for 1h. After successive rinsing with buffer and water, a (1:1) mixture of MCB and MPA (0.5 mM both, in water) was added for 1h. Thereafter, the modified substrate was rinsed with water and then with Tris-HCl buffer solution. DNA hybridization was conducted by exposing the ss-DNA modified surface to a solution of complementary flavin 5' ss-DNA (sequence: GTC ACG ATG GCC CAG TAG TT) (5 µM in buffer). The following experiments of nonbinding and binding dodecin incubation (5-10 µM in buffer, the protein solutions were filtered prior to use using a cellulose acetate filter, 0.2 µm Nalgene Nunc, International Corporation, New York) were carried out accordingly, and the modified surface was rinsed with buffer.

X-ray structural analysis. Crystallization of dodecin is described elsewhere.[3] Data on a dodecin holocomplex crystal (apododecin/CofC3_O5) were collected to 1.6 Å. Diffraction intensities were integrated with XDS and scaled with XSCALE[4]. An initial model (template 2ccc) was refined by cycles of model building in COOT[5] and automatic refinement in REFMAC[6] (see Table S1). Figures of crystal structures were created with PYMOL (http://pymol.org). In structures deposited to the pdb database, the atomic model of the ligand is reduced to the bound and non-flexible core structures. The atomic coordinates and structure factors of dodecin with bound ligand CofC3_O5 (code 4b2h) have been deposited in the Protein Data Bank, Research Collaboratory for Structural Bioinformatics, Rutgers University, New Brunswick, NJ (http://www.rcsb.org/).

Instrumentation.
Surface plasmon resonance spectroscopy (SPR). The SPR principle has been described in previous publications[7] and the setup is briefly described as follows. The setup is based on the Kretschmann configuration[8] used with a gold coating glass substrate, which is then optically matched to the base of a 90° glass prism. The plasmon surface polaritons are excited at the metal/dielectric interface, upon total internal reflection of the laser beam (HeNe, λ = 632.8 nm, power 5 mW) at the prism base. By adjusting the incident angles, the angle scan curve shows that there is a sharp minimum at the resonance angle which depends upon the precise architecture of the metal/dielectric interface. From the kinetic scan curve, the adsorption processes occurring at the interface can be followed in real time by selecting an appropriate incident angle and monitoring the reflectivity as a function of time.
Glass slides (n_{BK7} = 1.515, n_{LaSFN9} = 1.84489 at 633 nm), covered with a thin layer of titanium and gold, were optically coupled to a 90° prism of LaSFN9 (for LaSFN9 slides) or SF15, n = 1.69425 (for BK7 slides). For both setups index match of n = 1.7000 was used (CargilleLabs, Cedar Grove, NJ). For DNA as well as for the protein a refractive index of n = 1.5 was assumed.[9] SPR angle shifts were calculated using the Fresnel equations via SPR software (Winspall version 3.0.2.0, Max Planck Institute of Polymer Research, Mainz, Germany).
Electrochemistry. Combined electrochemical and SPR measurements were carried out in a home made flow cell with the Au substrate as working, a Pt foil as counter, and an Ag/AgCl coated Pt wire as pseudo-reference electrode. An EG&G potentiostat/galvanostat model 273 or 283 was used.
Atomic force microscopy (AFM). AFM investigations were made on the TSG slides, using an Asylum Research MFP-3D Bio (Asylum Research, Santa Barbara, California) operating in tapping mode with silicon probe tips (type RTESP, nominal resonance frequency 300 kHz, nominal spring constant 40 N/m) purchased from Veeco.

ii) Table S1. X-ray Data collection and refinement statistics

	dodecin with CofC3_O5 ligand (4b2h)
**Data collection**	
Space group	F 41 3 2
Cell dimensions□	*a*, *b*, *c* =142.00 Å
Resolution (Å)	50.0 – 1.6 (1.7-1.6)
R_{merge}	0.073 (0.625)
<*I* / □*I*>	31.04 (5.87)
Completeness (%)	99.9 (99.9)
Redundancy	23.3 (23.5)
**Refinement**	
Resolution (Å)	20.0 – 1.6
No. reflections	15863
R_{work} / R_{free}	0.167 / 0.178
No. atoms	
Protein	484
Waters	79
Ions	29
Ligands (SO_4^{2-}/ CofC3_O5)	5 / 25
Average *B*-factors	21.74
R.m.s deviations	
Bond lengths (Å)	0.033
Bond angles (°)	3.763
Ramachandran	
Most favored (%)	98.0
Additional allowed (%)	2.0

(Values in parentheses refer to highest-resolution shell)

iii) SPR long-term run (kinetic scan curve) depicted in Figure S1 including a detailed description of the individual experimental steps (shown previously as Figure 2C in the main manuscript)

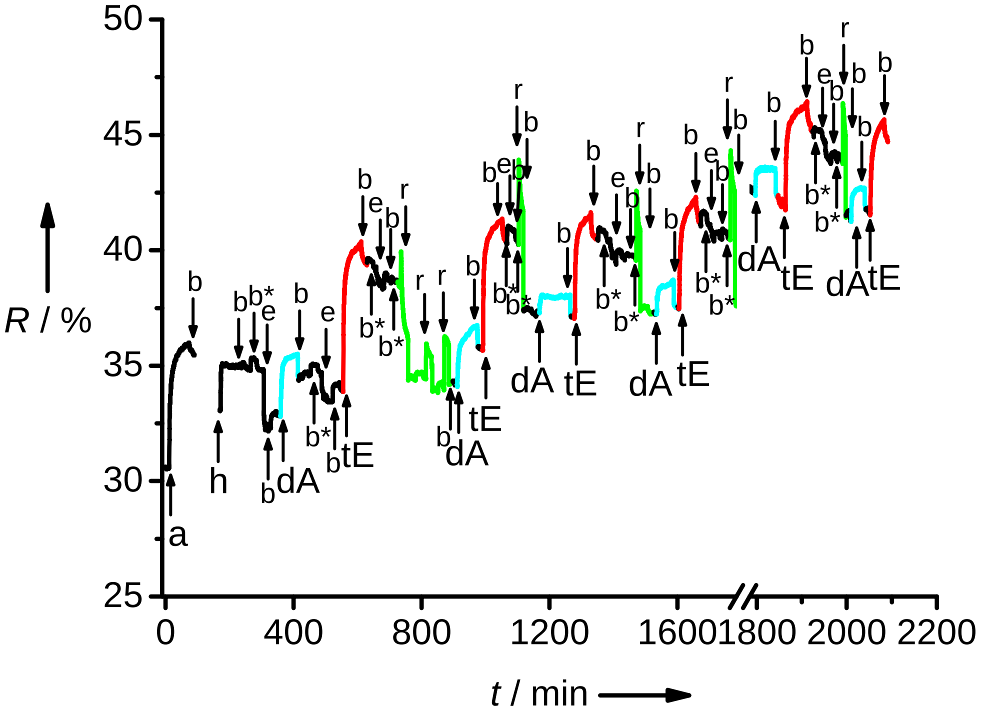

Figure S1. Kinetic SPR scan curve (long term run) including the following steps: adsorption of disulfide modified ss-DNA (a), rinsing with buffer (b), hybridization with flavin-modified ss-DNA (h), incubation of a non-binding apododecin variant as negative control (dA), reconstitution of dodecin by addition of high affinity binding apo-dodecin (tE) rinsing with argon saturated buffer (b*), electrochemistry, i.e. application of -550 mV vs. Ag/AgCl for 5 min followed by cyclic voltammetry (e), chemical reduction using sodium dithionite in argon saturated buffer solution (r). Incubation of dA/tE and rinsing with buffer is always shown in cyan/red, reduction and rinsing in green. In this long-term run the first chemical reduction was carried out in three steps (each with 20 mM sodium dithionite). Since a single reduction step with 20 mM sodium dithionite did not result in complete release of apododecin, the following chemical reductions were carried out as single reduction steps using 50 mM sodium dithionite.

iv) Optical thickness of the dodecin monolayers

From the angular scan curves (collected in between the individual surface modification steps shown in **Figure S1**) we calculated the optical thickness of the individual tE layers assuming a refractive index of $n = 1.5$ for dodecin (see **Table S2**).[9] While for the first dodecin layer a thickness of 1.8 nm was determined, the thickness of the sixth layer was still 0.9 nm (50% of the first). As it can be seen also from the increase in reflectivity throughout the experimental run, there was some aging of the system, but this might be a matter of further optimization.

Table S2. Optical thickness of the dodecin layers shown in **Figure S1**[a]

1st	2nd	3rd	4th	5th	6th
1.8 nm	1.6 nm	1.2 nm	1.3 nm	0.9 nm	0.9 nm

[a] calculated with a refractive index of $n = 1.5$.

References

[1] M. Grininger, G. Nöll, S. Trawöger, E.-K. Sinner, D. Oesterhelt, *Biointerphases* **2008**, *3*, 51-58.
[2] R. Naumann, S. M. Schiller, F. Giess, B. Grohe, K. B. Hartman, I. Kaercher, I. Koeper, J. Luebben, K. Vasilev, W. Knoll, *Langmuir* **2003**, *19*, 5435-5443.
[3] M. Grininger, K. Zeth, D. Oesterhelt, *J. Mol. Biol.* **2006**, *357*, 842-857.
[4] W. Kabsch, *J. Appl. Crystallogr.* **1988**, *21*, 67-71.
[5] P. Emsley, K. Cowtan, *Acta Crystallogr., Sect. D: Biol. Crystallogr.* **2004**, *D60*, 2126-2132.
[6] a) A. T. Brunger, P. D. Adams, G. M. Clore, W. L. DeLano, P. Gros, R. W. Grosse-Kunstleve, J.-S. Jiang, J. Kuszewski, M. Nilges, N. S. Pannu, R. J. Read, L. M. Rice, T. Simonson, G. L. Warren, *Acta Crystallogr., Sect. D: Biol. Crystallogr.* **1998**, *D54*, 905-921; b) T. A. Jones, J. Y. Zou, S. W. Cowan, M. Kjeldgaard, *Acta Crystallogr., Sect. A: Found. Crystallogr.* **1991**, *A47*, 110-119; c) G. N. Murshudov, A. A. Vagin, E. J. Dodson, *Acta Crystallogr., Sect. D: Biol. Crystallogr.* **1997**, *D53*, 240-255; d) B. Meissner, E. Schleicher, S. Weber, L.-O. Essen, *J. Biol. Chem.* **2007**, *282*, 33142-33154.
[7] W. Knoll, *Annu. Rev. Phys. Chem.* **1998**, *49*, 569-638.
[8] E. Kretschmann, *Z. Phys.* **1971**, *241*, 313-324.
[9] a) L. Duque, B. Menges, S. Borros, R. Foerch, *Biomacromolecules* **2010**, *11*, 2818-2823; b) A. Laschitsch, B. Menges, D. Johannsmann, *Appl. Phys. Lett.* **2000**, *77*, 2252-2254.
